# Improving detection of monogenic diabetes through reanalysis of *GCK* variants of uncertain significance

**DOI:** 10.1007/s00592-025-02449-8

**Published:** 2025-01-16

**Authors:** Sunita M. C. De Sousa, Jennifer M. N. Phan, Amanda Wells, Kathy H. C. Wu, Hamish S. Scott

**Affiliations:** 1 Adelaide Medical School, University of Adelaide, Adelaide, Australia; 2 Endocrine & Metabolic Unit, Royal Adelaide Hospital, Adelaide, Australia; 3 Adult Genetics Unit, Royal Adelaide Hospital, Adelaide, Australia; 4 Department of Genetics & Molecular Pathology, SA Pathology, Adelaide, Australia; 5 Flinders Medical School, Flinders University, Adelaide, Australia; 6 Clinical Genomics, St Vincent’s Hospital, Darlinghurst, Australia; 7 School of Medicine, University of New South Wales, Sydney, Australia; 8 Discipline of Genomic Medicine, Faculty of Medicine and Health, University of Sydney, Sydney, Australia; 9 School of Medicine, University of Notre Dame, Sydney, Australia; 10 Centre for Cancer Biology, an alliance between SA Pathology, University of South Australia, Adelaide, Australia

**Keywords:** Glucokinase, Monogenic diabetes, DNA sequencing, Genetics

## Abstract

**Aims:**

To assess the utility of reanalysing *GCK* variants of uncertain significance (VUS) as an intervention to improve the detection of monogenic diabetes.

**Methods:**

We examined *GCK* VUS in a local cohort of individuals with suspected monogenic diabetes and re-curated each variant against the recent ClinGen *GCK*-specific variant classification guidelines.

**Results:**

Variant reanalysis achieved a new ‘likely pathogenic’ classification (i.e., positive results) in 4/8 identified VUS. The single most common newly applied criterion indicating variant pathogenicity was a confirmed phenotype of *GCK*-hyperglycaemia. RNA sequencing and segregation studies were performed in two cases but not additive to reclassification.

**Conclusions:**

This is the first VUS reclassification study in monogenic diabetes using gene-specific guidelines. Within the limits of this small study, we observed a high rate (50%) of VUS upgrades to a positive result, thereby confirming the utility of VUS reanalysis– particularly with biochemical phenotyping– in increasing the detection of monogenic diabetes. We recommend HbA1c, fasting blood glucose and either pancreatic autoantibody negativity or a small oral glucose tolerance test increment as a feasible minimum dataset to inform variant classification at the individual patient level, noting the ongoing work of the ClinGen Monogenic Diabetes Expert Panel in systematically reviewing *GCK* variants at the international level.

## Introduction

The most prevalent form of monogenic diabetes is *GCK*-hyperglycaemia, where heterozygous loss-of-function variants in the *GCK* gene (encoding glucokinase) impede glucose sensing, leading to an isolated mildly raised glycaemic set-point that generally does not necessitate treatment or diabetes complication surveillance. Pregnancy is a special situation where insulin therapy may be used to mitigate macrosomia in an unaffected fetus. Despite the unequivocal value to individuals, families and healthcare systems in diagnosing *GCK*-hyperglycaemia, > 80% of monogenic diabetes cases remain undiagnosed [1].

In genetic testing, the American College of Medical Genetics and Genomics (ACMG) framework is used to classify identified variants as: benign, likely benign, variant of uncertain significance (VUS), likely pathogenic (LP) or pathogenic (P), with P/LP variants considered to be clinically actionable positive results and VUS regarded as inconclusive/negative results [2]. Classification is based on fulfilment of certain combinations of pathogenic vs. benign criteria of different strengths of certainty. One reason for missing a diagnosis of monogenic diabetes is when genetic testing only demonstrates a VUS with insufficient evidence to classify the variant as pathogenic (i.e., causative of hyperglycaemia). In VUS reclassification studies in other heritable conditions, most VUS remain as VUS or are downgraded to a benign classification [3]. Noting the recent development of international variant classification guidelines specific to *GCK* by The Clinical Genome (ClinGen) Resource, we reviewed all *GCK* VUS in a cohort of individuals with suspected monogenic diabetes to determine the rate and direction of VUS reclassification.

## Methods

We aimed to assess the utility of reanalysing *GCK* variants of uncertain significance (VUS) as an intervention to improve the detection of monogenic diabetes.

We searched genetic testing records in the SA Pathology Genetics & Molecular Pathology Laboratory in Adelaide, Australia to collate all previously identified VUS in *GCK*. Each VUS was then reviewed according to the ClinGen Monogenic Diabetes Variant Curation Expert Panel (VCEP) Specifications to the ACMG/AMP Variant Interpretation Guidelines for *GCK* Version 1.3.0 (https://cspec.genome.network/cspec/ui/svi/doc/GN086) as outlined in Table 1. Various combinations of specifications permit a VUS to be upgraded to LP classification, e.g., 2 moderate and ≥ 2 supporting criteria. Some specifications pertain to variant-specific information available online (e.g., genomic population databases), whilst other specifications pertain to patient-level data which were obtained through medical record review. Each specification has a standard level of evidence which may be upgraded or downgraded if additional criteria are fulfilled– e.g., a supporting ‘PP’ criterion may be upgraded to moderate in accordance with set rules, thereby making it a ‘PP_moderate’ criterion.

**Table 1:** Outline of ACMG variant classification as per ClinGen Monogenic Diabetes Variant Curation Expert Panel Specifications

Individual pathogenicity criteria		*GCK*-specific PP4 (Phenotype) criteria		Rules for combining criteria
PVS1	Null variant	Patient’s phenotype or family history is highly specific for a disease with a single genetic aetiology:		Pathogenic Variant PVS1 and • ≥ 1 PS or • ≥ 2 PM or • 1 PM + 1 PP or • ≥ 2 PP ≥ 2 PS 1 PS and • ≥ 3 PM or • 2 PM + ≥ 2 PP or • 1 PM and ≥ 4 PP
PS1	Same amino acid change	PP4_ Moderate	• HbA1c 5.6–7.6% (38–60 mmol/mol) AND • Fasting glucose 5.5–8 mmol/L (100–144 mg/dL) AND • Presence of any of the following additional features: • PP4 phenotype in paediatric patient AND • Not treated with insulin AND antibody negative • OR treated with insulin, antibody negative, and detectable C-peptide (> 0.6ng/mL) after 3 years • Multiple persistent values meeting above criteria or well-documented persistent IFG • OGTT with minimal increment < 3 mmol/l (54 mg/dl) • Antibody negative • Macrosomia in normoglycemic offspring of hyperglycemic gestational parent • Low birthweight in hyperglycemic offspring of hyperglycemic gestational parent • Three-generation, dominant family history of diabetes or hyperglycemia (in a family not used for PP1)	
PS2	*De novo* with confirmation of maternity/paternity + no FHx			
PS3	Functional studies			
PS4	Higher prevalence in affected individuals vs. controls			
PM1	Hotspot or functional domain without benign variation			
PM2	Absent from controls			
PM3	For AR, detected *in trans* with a pathogenic variant			
PM4	Protein length change due to in-frame del/ins			
PM5	Novel missense change at same codon			Likely Pathogenic Variant PVS1 + 1 PM 1 PS + 1 PM PVS1 + ≥ 1 PP 1 PS + 2 PM 1 PS + ≥ 2 PP ≥ 3 PM 2 PM + ≥ 2 PP 1 PM + ≥ 4 PP
PM6	Assumed *de novo* without confirmation of maternity/paternity			
PP1	Co-segregation with disease in multiple affected family members			
PP2	Missense variant in a gene that has a low rate of benign missense variation			
PP3	Computational evidence			
PP4	Phenotype (see next column)	PP4_ Supporting	• HbA1c 5.6–7.6% (38–60 mmol/mol) AND • Fasting glucose 5.5–8 mmol/L (100–144 mg/dL)	
***PP5***	*Reputable source (N/A)*			

The study was ethically approved by Central Adelaide Local Health Network Human Research Ethics Committee (HREC Ref no. 2021/HRE00232). Individuals with VUS that were upgradable to LP classifications were contacted to provide counselling regarding their newly positive genetic test results, and concurrently consented for study participation. The HREC waived the need for study consent in individuals whose VUS remained as VUS as patient re-contact was not otherwise required in these cases and only audit-level data are provided herein.

## Results

We identified eight individuals (age at testing 12–43 year, six females, two males) with previously identified *GCK* VUS on monogenic diabetes next generation sequencing (NGS) gene panel testing. Variant types comprised: four single nucleotide substitutions resulting in missense variants, one in-frame 3 bp deletion resulting in a single amino acid deletion, two small intronic variants, and one large multi-exon duplication, which is the first reported case of a duplication involving *GCK* [4]. Variant reclassification is outlined in Table 2, and comparisons between study curations and original curations are depicted in Table 3.

**Table 2:** VUS re-curation process and results of the patient cohort

	Case 1	Case 2	Case 3	Case 4	Case 5	Case 6	Case 7	Case 8
Demographics*	43yo female	22yo female	31yo female	17yo male	27yo female	12yo female	19yo female	38yo male
Variant type	Intronic insertion	Missense	Non-canonical splicing	Missense	Missense	In-frame amino acid deletion	Multi-exonic duplication	Missense
cDNA position†	c.209 − 12_209-11dup	c.397T > G	c.580–3 C > A	c.740 A > T	c.878T > C	c.1125_1127del	c.206 − 130_677-705dup	c.554T > C
Amino acid change	p.?	p.(Phe133Val)	p.?	p.(Asp247Val)	p.(Ile293Thr)	p.(Thr376del)	p.?	p.(Leu185Pro)
Year of original test	2021	2021	2022	2022	2023	2019	2019	2016
Criteria applied at original classification	PM2	PM2, PM5_PP, PP2, PP3	PS4_PP, PM2, PP1, PP3	PM2, PP3	PM2_PP, PP2, PP3, PP4	PM2	PM2	PM2, PP3
Criterial applied at re-curation	PM2_PP, PP4_M, BP4	PM2_PP, PP2, PP3	PM2_PP, PP1_M, PP3, PP4_M	PM2_PP, PP2	PS4_M, PM2_PP, PP2, PP3, PP4_M	PM2_PP, PM4_PP, PP4_M	PM1, PM2_PP, PM4_PP, PP4_M	PM2_PP, PP1_M, PP2, PP3, PP4_M
Additional patient assessments	Phenotyping (PP4_M)^**^	Phenotyping (did not meet PP4 criteria)^**^	Phenotyping (PP4_M): HbA1c 6.3%, fasting glucose 7.2 mmol/L, negative anti-GAD, IA2 and ZnT8 antibodies	Phenotyping (did not meet PP4 criteria)^**^	Phenotyping (PP4_M): HbA1c 6.3%, fasting glucose 6.7 mmol/L, negative anti-GAD, IA2 and ZnT8 antibodies, OGTT increment 0.9 mmol/L	Phenotyping (PP4_M)^**^; Segregation studies in 3 relatives with diabetes of uncertain type (all negative hence PP1 not applied)	Phenotyping (PP4_M): HbA1c 5.7%, fasting glucose 7.2 mmol/L, negative anti-GAD, IA2 and islet cell antibodies; RNA studies in blood indicated out-of-frame tandem duplication (but PS3 not applied as mRNA expression levels < quality threshold)	Phenotyping (PP4_M): HbA1c 6.9%, fasting glucose 6.0 mmol/L, negative anti-GAD and IA2 antibodies
Reproductive outcomes (in upgraded variant cases)	n/a^**^	n/a^**^	G1P1: insulin for pregnancy hyperglycaemia, birth weight 3270 g	n/a^**^	G1P2 (monozygotic twins): metformin for pregnancy hyperglycaemia, birth weights unknown	n/a^**^	G1P1: insulin for pregnancy hyperglycaemia, birth weight 3500 g	Male, 2 children, birth weights unknown
Re-curation conclusion	Remains VUS	Remains VUS	Upgraded to LP	Remains VUS	Upgraded to LP	Remains VUS	Upgraded to LP	Upgraded to LP

*at time of initial testing; **data not provided to protect patient anonymity– the need for consent was waived in cases where the VUS was not upgraded and hence the patient was not re-contacted; †RefSeq: NM_000162.5

### Individual pathogenicity criteria

Wherever the PM2 criterion (absent from controls) was applied in the original classification, we downgraded the criterion from moderate to supporting (i.e., ‘PM2_supporting’) in line with *GCK* VCEP recommendations (Table 3).

**Table 3:**
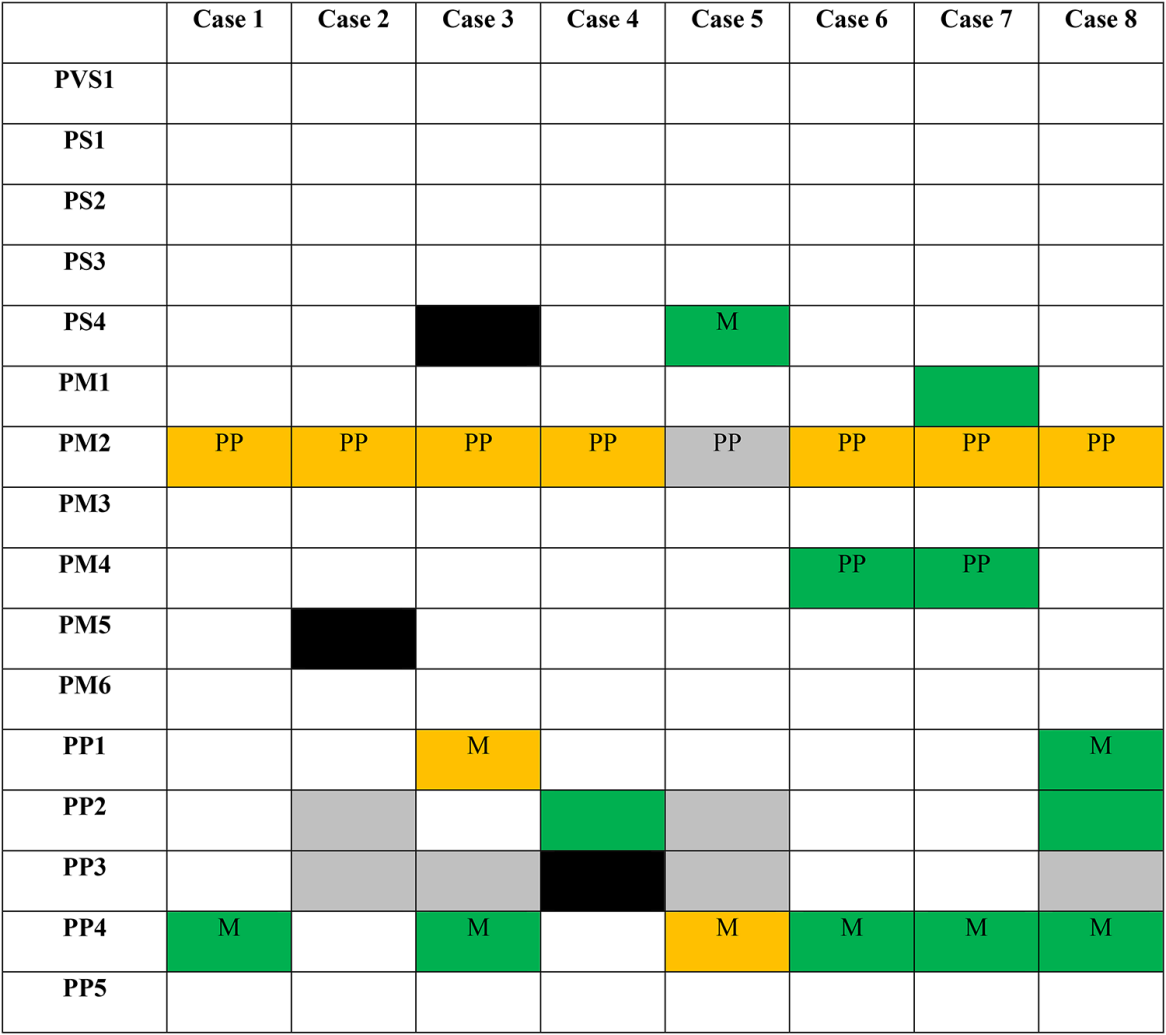
Applicable variant pathogenicity criteria in the patient cohort at original vs. study curation

LP, likely pathogenic; M, moderate; PM, pathogenic moderate; PP, pathogenic supporting; PS, pathogenic strong; PVS, pathogenic very strong; VUS, variant of uncertain significance

Legend: green = new criteria, orange = modified criteria, grey = retained criteria, black = removed criteria; criteria with strength modifications are indicated by box text

Three other pathogenicity criteria were modified or newly applied at moderate strength. The most common newly applied criterion was the PP4 criterion (specific phenotype), which was based on review of pre-existing glycohaemoglobin, blood glucose and pancreatic autoantibody results, with a moderate level of evidence achieved in each applicable case. By the *GCK* VCEP guidelines, the PP4_moderate criterion is applicable when an individual has an HbA1c of 5.6–7.6% (38–60 mmol/mol) (maximum value used if multiple results) AND fasting glucose 5.5–8 mmol/L (100–144 mg/dL) AND the presence of an additional feature of *GCK*-hyperglycaemia as outlined in Table 1 (e.g., negative pancreatic autoantibodies, oral glucose tolerance test with minimal increment < 3 mmol/l (54 mg/dl) etc.). If only HbA1c and fasting glucose criteria are met without additional *GCK*-hyperglycaemia features, then PP4 is applicable at its usual supporting level of pathogenicity. In our cohort, PP4 had only been applied in 1/8 original classifications (Case 5), and only at a supporting level of evidence, in a case where the referring clinician specified that the phenotype was suspicious of *GCK*-hyperglycaemia. On variant review for the study, we were able to apply PP4 in this case and an additional 5 cases by extracting patient data and appraising it against the *GCK* VCEP specifications for PP4. In each of these 6 cases, there was evidence to fulfil the moderate version of the PP4 criterion based on HbA1c 5.6–7.6%, fasting glucose 5.5–8 mmol/L and negative pancreatic autoantibodies.

Another newly applied criterion of moderate strength was the moderate version of the PS4 criterion (prevalence in affected individuals vs. controls), which was fulfilled in Case 5 based on the finding of the variant in published literature (3 occurrences) and another Australian laboratory (1 occurrence). As the PS4_moderate criterion requires 4–6 occurrences of a variant in affected unrelated individuals, the other Australian laboratory observation of this variant was essential to achieve this pathogenicity criterion. It was identifiable due to an entry in the Shariant database, which is an online platform that shares variant interpretations and associated evidence between Australian clinical genetic testing laboratories [5].

The remaining newly applied criterion of moderate strength was PM1 (altered protein length), applied to the multi-exon *GCK* duplication. RNA studies were attempted in this case to secure the higher level pathogenicity criterion of PS3 (in vivo/in vitro functional study). Reverse transcriptase-PCR (RT-PCR) using a fresh blood sample from the patient indicated tandem duplication of exons 3–6, resulting in a frameshift and premature termination codon, but the level of mRNA expression in blood did not meet the quality threshold for this assay and was therefore not used for variant classification. Furthermore, the *GCK* VCEP specifications preclude the use of patient cell lines in RNA evidence, with the presumption that there might be a different occult variant in the individual that is causing aberrant splicing rather than the variant in question.

Segregation studies were attempted to achieve the PP1 criterion in Case 6 but all available relatives with diabetes (unclear if *GCK*-hyperglycaemia phenotype) tested negative for the proband’s VUS.

### Overall variant classification

Ultimately, we were able to upgrade 4/8 (50%) VUS (i.e., uninformative genetic test results) to an LP classification (i.e., positive results). This comprised 4/6 cases with the PP4_moderate criterion (including Case 5 with the PS4_moderate criterion and Case 7 with the PM1 criterion), whilst the other 2/6 cases had insufficient additional criteria to reach LP classification. Cascade testing of relatives with hyperglycaemia is currently underway.

## Discussion

To our knowledge, this is the first VUS reclassification study in monogenic diabetes using the ClinGen gene-specific framework. We achieved a high rate (50%) of VUS reclassification to positive results without the need for additional tests. Two prior studies utilising the generic ACMG variant classification guidelines with intensive functional experiments also achieved high rates of VUS reclassification in the monogenic diabetes setting, but with a substantially greater burden of investigation that is unfeasible in routine practice [6, 7]. The high VUS upgrade rates of 50–74% between these monogenic diabetes studies far exceed rates in non-diabetes conditions [3, 8, 9]. Why there is a higher yield of VUS reclassification in diabetes is uncertain but may relate to endocrine genes being less studied than other conditions (e.g., cancer, deafness), resulting in a greater burden of true disease-causing variants classified as VUS due to a lack of previous observations in the literature/patient databases that would permit pathogenic classification.

The pathogenicity criterion of greatest utility in VUS reclassification was a clinical phenotype specific for *GCK*-hyperglycaemia, emphasising the importance of referring clinicians carefully assessing the patient’s glycaemic history to evaluate the pre-test probability of finding a LP/P *GCK* variant and providing this information on request forms to aid the genetic testing laboratory in variant classification. Based on our experience in the current study, we recommend noting HbA1c, fasting blood glucose and either antibody negativity or a small oral glucose tolerance test (OGTT) increment as a feasible minimum dataset to achieve the phenotype criterion at a moderate degree of pathogenicity. Detailed phenotyping to the strength required for a moderate pathogenicity criterion for other genes that currently do not have gene-specific VCEP guidance may be facilitated by multidisciplinary variant review boards with involvement of the treating clinician and reporting laboratory [10]. In Australia, the EndoGen National Endocrine Genetics Network holds regular national multidisciplinary team meetings involving endocrinologists, clinical geneticists, genetic counsellors, genetic pathologists and genetic scientists, and multiple VUS reclassifications have been achieved through this forum.

Another useful pathogenicity criterion was comparing the rate of the variant in affected individuals vs. healthy controls. This may be achieved by inter-laboratory liaison, most efficiently via centralised databases (e.g., the Australian Shariant database) that facilitate case and variant classification sharing between participating laboratories [5].

VUS that potentially affect splicing are particularly difficult to assess. In general terms, aberrant splicing may be elucidated through RNA studies; however, *GCK* is poorly expressed in blood, making it difficult to perform RNA expression studies using blood samples. Furthermore, the *GCK* VCEP specifications explicitly exclude RNA studies from patient cell lines from being used as a strong pathogenicity criterion due to the possibility that the resultant splicing may relate to other unidentified genomic variation. A supporting criterion based on in silico prediction of aberrant splicing may instead be used; this was used to support pathogenicity for one intronic variant with a maximum SpliceAI score > 0.2 (Case 3) and to support ‘benignity’ of the other intronic variant with SpliceAI scores < 0.2 (Case 1). An alternative approach is to perform in vitro functional splicing assays via a minigene construct after prioritisation of putative splicing variants using in silico models [7]; however, this is a labour-intensive process that is generally not feasible in individual patient care.

How VUS are managed is of increasing importance given the rise of gene panel testing which expectedly produces a greater burden of VUS than single gene sequencing. The use of gene-specific guidelines should, as in the present study, bring clarity to many variants otherwise considered to be of uncertain significance. This may be of particular value in individuals where gene panel testing reveals variants in multiple genes. Lucchesi et al. described one such kindred where dual variants were found in *GCK* and *HNF1A*, with the *GCK* variant ultimately classified as pathogenic and the *HNF1A* classified as a VUS through ClinGen Monogenic Diabetes Expert Panel guidance in conjunction with the family’s phenotype compatible with *GCK*-hyperglycaemia [11].

Aside from unresolved VUS, another potential cause of a false-negative genetic test result in individuals with monogenic diabetes is missed copy number variants (CNV). These variants typically necessitate dedicated copy number analysis to be performed, either via whole exome sequencing (WES)-based CNV-calling bioinformatic pipelines as routinely employed in our laboratory [12], or via multiplex ligation-dependent probe amplification (MLPA) [13]. Our cohort includes the first reported duplication involving *GCK*. By contrast, multiple partial or whole *GCK* deletions have been previously reported. As highlighted in a report by Yu et al., *GCK* deletions may be missed not only by Sanger sequencing but also by WES unless dedicated CNV detection using WES data is pursued [13]. These cases emphasise the importance of considering CNVs– including duplications– in the assessment of suspected monogenic diabetes.

The major limitation of our study is its small size. We acknowledge that the ClinGen VCEP that created the *GCK*-specific guidelines is charged with systematic consensus review of monogenic diabetes gene variants for pathogenicity and submission to the ClinVar database. This is an ongoing consolidated program of variant reanalysis by many internationally recognised experts that will clarify the pathogenicity of a vast number of VUS in the coming years. Our present work is complementary to the aims of the ClinGen VCEP in its immediate, real-world application of the *GCK*-specific VCEP guidelines, which has already resulted in the revision of our patients’ diagnoses to *GCK*-hyperglycaemia. This study, albeit small, should raise awareness amongst local laboratories of the option of immediate VUS reanalysis using the *GCK*-specific VCEP guidelines. For treating clinicians, this study highlights the utility of VUS reanalysis in the diagnostic work-up of individual patients and the importance of collating the required patient-level data. The timing of VUS reanalysis is especially important in reproductive age women given the significant impact on management from a diagnosis of *GCK*-hyperglycaemia rather than gestational diabetes. Noting that many ClinGen VCEPs exist at various levels of progress across the specialty of clinical genetics, clinicians and laboratories should routinely consider, at least, the option of reanalysis of reported VUS rather than waiting for VCEP outcomes which will understandably take time given the complexity of the task and may not examine the particular VUS in question. Additionally, although desirable, local laboratories may not necessarily have a mechanism by which to flag VUS that have been upgraded by a VCEP and so clinician request for VUS reanalysis remains an important clinical tool for patients in the community.

In conclusion, this study confirms the utility of VUS reclassification using gene-specific VCEP guidelines in monogenic diabetes. Readily available biochemical phenotyping data were instrumental in reclassification, whilst the more intensive processes of RNA studies and segregation studies performed here were not directly additive. How we assess *GCK* variants will be of increasing importance given recent recommendations for more widespread *GCK* testing e.g., in all non-obese women with gestational diabetes [14]. The 50% rate of upgraded VUS is especially striking given that a diagnosis of *GCK*-hyperglycaemia typically allows cessation of diabetes treatment and surveillance. Pending further studies, there may be a cost-effectiveness argument for routine *GCK* VUS reclassification with careful biochemical phenotyping to avoid ongoing costs of unnecessary diabetes care. Looking forward, clinicians should ideally provide the relevant biochemical data to laboratories at the time of genetic test requesting to allow accurate upfront variant classification and avoid the burden of VUS for patients, clinicians and laboratories.

## Data Availability

Restrictions apply to the availability of data generated and analysed during this study to preserve patient confidentiality. The corresponding author will on request detail the restrictions and any conditions under which access to some data may be provided.

## Abbreviations

PVS: Pathogenic very strong
PS: Pathogenic strong
PM: Pathogenic moderate
PP: Pathogenic supporting
FHx: Family history
AR: Autosomal recessive
del/ins: Deletion/insertion
IFG: Impaired fasting glucose
OGTT: Oral glucose tolerance test

## References

[1] Shields BM, Hicks S, Shepherd MH, Colclough K, Hattersley AT, Ellard S (2010) Maturity-onset diabetes of the young (MODY): how many cases are we missing? Diabetologia 53:2504–2508 20499044 10.1007/s00125-010-1799-4

[2] Richards S, Aziz N, Bale S, Bick D, Das S, Gastier-Foster J et al (2015) Standards and guidelines for the interpretation of sequence variants: a joint consensus recommendation of the American College of Medical Genetics and Genomics and the Association for Molecular Pathology. Genet Med 17:405–423 25741868 10.1038/gim.2015.30 PMC4544753

[3] SoRelle JA, Thodeson DM, Arnold S, Gotway G, Park JY (2019) Clinical utility of reinterpreting previously reported genomic Epilepsy Test results for Pediatric patients. JAMA Pediatr 173:e182302 30398534 10.1001/jamapediatrics.2018.2302 PMC6583457

[4] De Sousa SMC, Wu KHC, Colclough K, Rawlings L, Dubowsky A, Monnik M et al (2024) Identification of monogenic diabetes in an Australian cohort using the Exeter maturity-onset diabetes of the young (MODY) probability calculator and next-generation sequencing gene panel testing. Acta Diabetol 61:181–188 37812285 10.1007/s00592-023-02193-x PMC10866744

[5] Tudini E, Andrews J, Lawrence DM, King-Smith SL, Baker N, Baxter L et al (2022) Shariant platform: enabling evidence sharing across Australian clinical genetic-testing laboratories to support variant interpretation. Am J Hum Genet 109:1960–1973 36332611 10.1016/j.ajhg.2022.10.006 PMC9674965

[6] Kavitha B, Ranganathan S, Gopi S, Vetrivel U, Hemavathy N, Mohan V et al (2023) Molecular characterization and re-interpretation of HNF1A variants identified in Indian MODY subjects towards precision medicine. Front Endocrinol (Lausanne) 14:1177268 37396188 10.3389/fendo.2023.1177268 PMC10313120

[7] Bouvet D, Blondel A, de Sainte Agathe J-M, Leroy G, Saint-Martin C, Bellanné-Chantelot C (2023) Evaluation in monogenic diabetes of the impact of *GCK*, *HNF1A*, and *HNF4A* variants on splicing through the combined use of in Silico Tools and Minigene assays. Hum Mutat 2023:6661013 40225161 10.1155/2023/6661013 PMC11919142

[8] Kim SY, Kim BJ, Oh DY, Han JH, Yi N, Kim NJ et al (2022) Improving genetic diagnosis by disease-specific, ACMG/AMP variant interpretation guidelines for hearing loss. Sci Rep 12:12457 35864128 10.1038/s41598-022-16661-x PMC9304357

[9] Chiang J, Chia TH, Yuen J, Shaw T, Li ST, Binte Ishak ND et al (2021) Impact of variant reclassification in Cancer Predisposition genes on Clinical Care. JCO Precis Oncol 5:577–584 34994607 10.1200/PO.20.00399

[10] Ellard S, Baple EL, Callaway A, Berry I, Forrester N, Turnbull C et al (2020) ACGS best practice guidelines for variant classification in rare disease version 4.01

[11] Lucchesi D, Randazzo E, Del Prato S, Bianchi C (2024) An Italian MODY family with proband and son carrying variants in GCK and HFN1A: is it a true case of digenic MODY? Acta Diabetol 61:131–134 37730861 10.1007/s00592-023-02171-3

[12] De Sousa SMC, Toubia J, Hardy TSE, Feng J, Wang P, Schreiber AW et al (2020) Aberrant splicing of SDHC in families with unexplained succinate dehydrogenase-deficient paragangliomas. J Endocr Soc 4:bvaa071 33195952 10.1210/jendso/bvaa071 PMC7646550

[13] Yu R, Zhang H, Xiao X (2024) Partial GCK gene deletion mutations causing maturity-onset diabetes of the young. Acta Diabetol 61:107–115 37704826 10.1007/s00592-023-02173-1

[14] Murphy R, Colclough K, Pollin TI, Ikle JM, Svalastoga P, Maloney KA et al (2023) The use of precision diagnostics for monogenic diabetes: a systematic review and expert opinion. Commun Med 3:136 37794142 10.1038/s43856-023-00369-8 PMC10550998

