# Correction: Improving detection of monogenic diabetes through reanalysis of *GCK* variants of uncertain significance

**DOI:** 10.1007/s00592-025-02467-6

**Published:** 2025-02-27

**Authors:** Sunita M. C. De Sousa, Jennifer M. N. Phan, Amanda Wells, Kathy H. C. Wu, Hamish S. Scott

**Affiliations:** 1 Adelaide Medical School, University of Adelaide, Adelaide, Australia; 2 Endocrine & Metabolic Unit, Royal Adelaide Hospital, Adelaide, Australia; 3 Adult Genetics Unit, Royal Adelaide Hospital, Adelaide, Australia; 4 Department of Genetics & Molecular Pathology, SA Pathology, Adelaide, Australia; 5 Flinders Medical School, Flinders University, Adelaide, Australia; 6 Clinical Genomics, St Vincent’s Hospital, Darlinghurst, Australia; 7 School of Medicine, University of New South Wales, Sydney, Australia; 8 Discipline of Genomic Medicine, Faculty of Medicine and Health, University of Sydney, Sydney, Australia; 9 School of Medicine, University of Notre Dame, Sydney, Australia; 10 Centre for Cancer Biology, an alliance between SA Pathology, University of South Australia, Adelaide, Australia

**Correction: Acta Diabetologica** 10.1007/s00592-025-02449-8

In the original version of this article, Table 3 was inadvertently published in black and white instead of colour; Table 3 should have appeared as shown below.

**Table 3:**
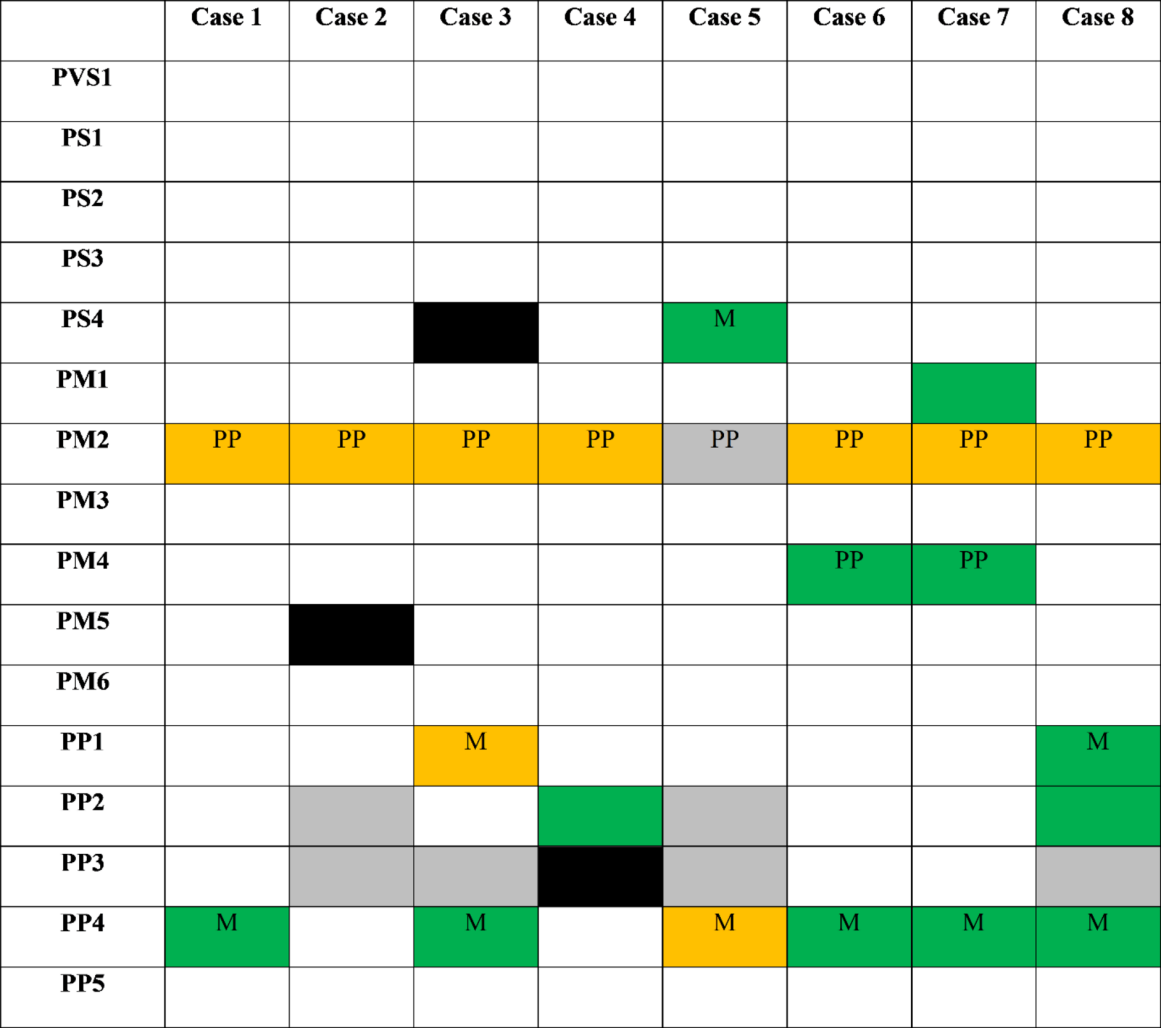
Applicable variant pathogenicity criteria in the patient cohort at original vs. study curation

LP, likely pathogenic; M, moderate; PM, pathogenic moderate; PP, pathogenic supporting; PS, pathogenic strong; PVS, pathogenic very strong; VUS, variant of uncertain significance

Legend: green = new criteria, orange = modified criteria, grey = retained criteria, black = removed criteria; criteria with strength modifications are indicated by box text

The original article has been corrected.

